# GC-MS and GC-IMS Based Metabolomics Combined with Cellular Assays to Characterize Volatile Compounds and Pharmacological Activity of *Lysimachia foenum-graecum* Hance from Different Origins

**DOI:** 10.3390/foods15122245

**Published:** 2026-06-22

**Authors:** Yu Li, Yunhao Zhao, Tao Jiang, Yuanpeng Hao, Junsheng Tian

**Affiliations:** 1 Modern Research Center for Traditional Chinese Medicine, Shanxi University, No. 92, Wucheng Road, Taiyuan 030006, China; 15536834028@163.com (Y.L.); zyh1551008237@sina.com (Y.Z.); 18235029274@163.com (T.J.); haoyuanpeng@sxu.edu.cn (Y.H.); 2 Key Laboratory of Research and Utilization of Bioactive Components in Famous Shanxi Medicinal Materials, No. 92, Wucheng Road, Taiyuan 030006, China; 3 Biomedical and Health Laboratory in Shanxi Province, No. 92, Wucheng Road, Taiyuan 030006, China

**Keywords:** *Lysimachia foenum-graecum* Hance, different origin, GC-IMS, GC-MS, aroma characteristics analysis, neuroprotective activity

## Abstract

*Lysimachia foenum-graecum* Hance (LFG), renowned for its potent aroma, is widely utilized as a culinary spice in the food industry. This study employed GC-IMS and GC-MS to analyze samples from four distinct regions: Guangxi (GX), Guizhou (GZ), Sichuan (SC), and Yunnan (YN). The profiling effort led to the identification of 51 and 47 volatile compounds, respectively, with alcohols, aldehydes, esters, and ketones constituting the predominant organic compounds (74%). The GX samples exhibited the highest overall compound content, while SC samples were characterized by the highest ester content. Significant variations in compound concentrations were observed across different sources. PLS-DA identified 15 distinctive volatile markers that differentiate the four regions. Odor activity value (OAV) analysis revealed five key volatile compounds linalool, borneol, estragole, undecanal, and β-Ionone. In addition, cellular-level assays demonstrated the pharmacological activity of LFG. These findings provide valuable insights for the application of LFG in the food industry.

## 1. Introduction

*Lysimachia foenum-graecum* Hance (LFG) is a perennial herbaceous plant primarily found in southern China. When the stems and leaves are dried, they emit a distinctive and long-lasting aroma, which can be directly utilized in cooking to enhance flavor and mask undesirable odors. It is commonly employed in the preparation of stir-fried meat dishes, hot pot seasonings, and preserved foods. Additionally, it can be processed into essential oils for use in the fragrance and tobacco industries and serves as a key aromatic ingredient in Zhuyeqing Liquor, contributing to its unique bouquet [1]. Beyond its culinary applications, LFG is also employed in traditional Chinese medicine is indicated for a range of ailments from common colds, headaches, and sore throats to toothaches, abdominal pain, and intestinal parasitosis [2]. Furthermore, the antioxidant activity of its extract is closely linked to its neuroprotective properties [3], indicating promising applications in both healthcare and the food industry.

In daily life, the aroma is one of the key factors influencing the quality of LFG and consumer preferences. Volatile compounds (VOCs) are the main components that influence the aroma characteristics of plants [4]. These aroma-active compounds like terpenes, alcohols, aldehydes, esters, and ketones contribute directly to the sensory experience perceived by consumers [5]. To identify the key VOCs that contribute to the overall aroma characteristics, the odor activity value (OAV), defined as the ratio of a compound’s concentration to its odor threshold, is a key metric for assessing the contribution of individual volatiles to the overall aroma [6]. OAV represents the relationship between a volatile compound’s concentration and its perceptual odor threshold (OT). Compounds with an OAV ≥ 1 are generally regarded as significant contributors to the overall flavor [7], thereby enabling the identification of key aroma compounds.

In order to further explore whether there are differences among different origins and to study the aroma characteristic components of LFG, we adopted the combined analysis of gas chromatography-mass spectrometry (GC-MS) and gas chromatography-ion mobility spectrometry (GC-IMS) to analyze the volatile components. This is also a commonly used analytical method in the food field in recent years [8], which can be used to distinguish rice from different sources and conduct a chemical composition study of Star anise [9,10]. GC-IMS combines the high separation efficiency of gas chromatography with the rapid response of ion mobility spectrometry, featuring fast separation and high sensitivity [11]. It also does not require complex sample preparation and can better preserve the original aroma characteristics of LFG. These advantages make GC-IMS of great value in the study of food aroma characteristics [12]. Leveraging the complementary strengths of both methods enables the rapid and precise determination of the VOCs in LFG [13]. The integration of multiple analytical techniques yields a more comprehensive, reliable, and scientifically robust understanding of the data [14].

Therefore, the aim of this study was to investigate the influence of different geographical origins (Guangxi, Guizhou, Sichuan, and Yunnan) on the volatile organic compounds (VOCs) of LFG using GC-MS and GC-IMS combined with multivariate statistical analysis. Key aroma-active VOCs were to be screened based on their odor activity values (OAVs). Furthermore, this study sought to evaluate the pharmacological activity of LFG using a PC12 cell injury model, specifically examining its effects on BDNF and p-Akt1 levels. The ultimate goal was to provide a theoretical basis for understanding the differences among LFG from the four production areas and to offer reference value for its application development and quality control.

## 2. Materials and Methods

### 2.1. Materials and Chemicals

LFG samples were collected from Jinxiu Yao Autonomous County, Guangxi Zhuang Autonomous Region (LatLng 24.12929, 110.19079); Leigong Mountain area, Guizhou Province (LatLng 26.38385, 108.07745); Meigu County, Sichuan Province (LatLng 28.32596, 103.13116); and Lincang City, Yunnan Province (LatLng 23.14821, 99.24545). All samples were identified as *Lysimachia foenum-graecum* Hance by Professor Xuemei Qin of Shanxi University. LFG samples were naturally shade-dried in cool, ventilated, dry places without direct sunlight. The dried whole herbs were ground into powder and stored at 4 °C for subsequent analysis. Six replicates were collected from each region.

Acetic acid, methyl acetate, methyl linoleate, ethanol, cineole, linalool, n-alkanes C7–C40, and 3-octanol were purchased from Chengdu Push Bio-technology Co., Ltd. (Chengdu, China). n-Hexane (analytical grade) was from Tianjin Damao Chemical Reagent Factory (Tianjin, China). PC12 cells were provided by the Cell Bank of the Chinese Academy of Sciences (Shanghai, China). RPMI 1640 medium, fetal bovine serum, and penicillin-streptomycin were from Hyclone (Logan, UT, USA). Trypsin-EDTA digestion solution was obtained from Solarbio (Beijing, China). Cell Counting Kit-8 was purchased from APExBIO Technology LLC (Houston, TX, USA). Rat p-Akt1 ELISA Kit and rat BDNF ELISA Kit were from Shanghai Aimeng Youning Biotechnology Co., Ltd. (Shanghai, China).

### 2.2. Distillation of LFG Essential Oil for GC-MS Analysis

The extraction of volatile compounds from LFG was performed using a Clevenger-type apparatus [15]. Briefly, 15 g of LFG was placed in a round-bottom flask with 300 mL of distilled water, and 50 µL of 3-octanol (0.2 ng/mL) was added as an internal standard. A Clevenger trap filled with distilled water was connected to the top of the flask, and 1 mL of n-hexane was layered on the water surface. After hydrodistillation for 3 h, the n-hexane layer containing the volatiles was collected and adjusted to 1 mL with n-hexane prior to GC analysis.

### 2.3. Sensory Analysis

The members of the sensory evaluation team were composed of master’s degree students from Shanxi University. There were 7 males and 3 females, all of whom were well-trained. The sensory evaluation was conducted on dried LFG samples (without any pretreatment or addition of water). According to the Chinese National Standard for Sensory Analysis (GB/T 10221-2021) [16], they sought aroma terms to describe the aroma characteristics and finally determined five descriptions: herbal, sweet, fresh, spicy, and woody. Then, they scored the LFG aroma. The team members evaluated the intensity of the odor attributes in each sample using a 10-point intensity scale (0–5, with an increment of 0.5, where 0 and 5 correspond to no and very strong respectively). Each sample was evaluated in triplicate, and the final result was provided based on the average score given by the 10 team members and plotted on a radar chart.

### 2.4. Characterization of Volatile Compounds in LFG by GC-MS

The volatile compounds were analyzed directly from dried LFG samples using an Agilent 8890 gas chromatograph coupled to a 7000D mass spectrometer (Agilent Technologies, Palo Alto, CA, USA). Separation was performed on a DB-17 MS capillary column (30 m × 0.25 mm × 0.25 μm). Helium was used as the carrier gas at a constant flow rate of 1 mL/min, with a splitless injection. The analysis was conducted under the following conditions: injection volume, 1 μL; injector and MS transfer line temperatures, 280 °C each. The GC oven was programmed as follows: initial temperature 40 °C, increased to 240 °C at 5 °C/min and held for 3 min, then raised to 250 °C at 5 °C/min and held for 3 min. Mass spectrometric detection was performed with electron ionization (EI) at 70 eV; the ion source temperature was 250 °C, and the mass range was set to 50–550 amu with a 3 min solvent delay.

The acquired data were processed using Agilent Qualitative Navigator software (B.06.00). Peaks with a relative peak area exceeding 1% were selected and their integration results were exported. Volatile compounds were identified by matching their mass spectra with those in the NIST 17 database. The identifications were further confirmed by comparing calculated Retention Indices (RIs) with the literature values, where RIs were determined through the analysis of a C7–C40 n-alkane series under identical chromatographic conditions.

The semi-quantitative determination of the compound was carried out using internal standard 3-octanol (1 μg/L).

### 2.5. Characterization of Volatile Compounds in LFG by GC-IMS

The volatile compounds were analyzed directly from dried LFG samples. The GC-IMS setup consisted of a FlavourSpec^®^ GC-IMS instrument (G.A.S, Dortmund, Germany) equipped with a DB-WAX capillary column (15 m × 0.52 mm × 1 μm). A 1 g LFG sample powder was placed in a 20 mL headspace vial and incubated at 65 °C for 20 min with an agitation speed of 500 rpm. Then, 500 μL of headspace gas was injected into the inlet using a heated syringe at 80 °C. The carrier gas/drift gas was N_2_. The carrier gas flow rate was programmed as follows: 0 to 2 min, 2 mL/min; 2 to 10 min, 10 mL/min; 10 to 20 min, 100 mL/min; 20 to 30 min, 150 mL/min; 30 to 40 min, 150 mL/min. The column temperature was maintained at 60 °C. The drift tube temperature was set at 40 °C with a drift gas flow rate of 150 mL/min.

Data analysis was carried out with the GC-IMS Library Search V2.2.1 software. Volatile compounds were identified by matching their experimentally derived Retention Indices (RIs, calculated using n-ketones C4–C9 as external references) and drift times against the NIST and IMS databases. The reporter plugin was used to generate three-dimensional and two-dimensional spectra, as well as comparative plots between samples. Fingerprint chromatograms were generated using the Gallery Plot plugin.

### 2.6. Quantification and Calculation of Odor Activity Values (OAV)

The aroma emitted by substances is usually related to the volatile compounds they contain [17], and the aroma activity value is an important indicator for evaluating the contribution of volatile compounds to the overall aroma of the substance. Generally, compounds with an OAV > 1 are considered key aroma compounds [18]. The calculation formula is as follows:

OAV = C/T

In the equation provided, C represents the concentration of volatile flavor compounds (µg/kg), while T denotes the odor threshold value of volatile flavor material in the water (µg/L).

### 2.7. Establishment of a Corticosterone-Induced PC12 Cell Injury Model and Cell Proliferation-Cytotoxicity Assay

PC12 cells in the logarithmic growth phase were seeded at 1 × 10^5^ cells per well in a 96-well plate. Each well was added to with 100 µL of complete medium. After 24 h of culture, cells were divided into a control group, a blank group, and five groups treated with different concentrations of corticosterone (CORT) (100 μmol/L, 200 μmol/L, 300 μmol/L, 400 μmol/L, 500 µmol/L) to establish six replicate wells. After treatment, cells were cultured for 24 h at 37 °C in a 5% CO_2_ incubator. Supernatant was removed, and 100 µL complete medium plus 10 µL CCK-8 working solution were added. Incubate for 2 h, gently shake for 1 min, then measure at 450 nm wavelength using an enzyme-linked immunosorbent assay reader. Cell survival rate was calculated as: (OD (experimental group)—OD (blank well)/OD (control group)—OD (blank well)) × 100%.

For the cytotoxicity assay, PC12 cells were seeded in 96-well plates at a density of 1 × 10^5^ cells per well and treated with LFG extract at concentrations of 25 µg/mL, 50 µg/mL, 100 µg/mL, 200 µg/mL, and 400 µg/mL. After 24 h of incubation, the cell survival rate was calculated.

### 2.8. Expression of Brain-Derived Neurotrophic Factor (BDNF) and Phosphorylated Akt1 (p-Akt1)

The BDNF and p-Akt1 kits were used for detection. Cell treatment: cells in the logarithmic growth phase were plated in 6-well plates at a density of 1 × 10^6^ cells per well and assigned to one of three groups: blank, model, and treatment. Each group was set up in triplicate, and the plates were subsequently maintained in an incubator for 24 h. After cell attachment, 2 mL of culture medium containing CORT was added to each well. After 24 h, the supernatant culture medium was discarded and replaced with culture medium containing LFG. The cells were cultured for another 24 h. Then, the cell suspension was diluted with PBS to reach 100 million/mL. The cells were repeatedly frozen and thawed to disrupt the cells and release intracellular components. The supernatant was collected after centrifugation at 4500 r for 20 min and used for the experiment.

### 2.9. Statistical Analysis

Six replicate samples were analyzed for each origin. Volatile compound contents were calculated in Excel, with results expressed as mean ± standard deviation (SD). Bubble stacked plots, percentage composition plots, and histograms were developed using ChipPlot “https://www.chiplot.online/index.html (accessed on 1 March 2026)”. Heatmaps were performed using the Metware Cloud “https://cloud.metware.cn (accessed on 4 March 2026)”. ANOVA and partial least squares-discriminant analysis (PLS-DA) were performed using MetaboAnalyst 6.0 “https://www.metaboanalyst.ca/ (accessed on 7 March 2026)”, with variable importance in projection (VIP) values calculated.

## 3. Results and Discussion

### 3.1. GC–IMS Topographic Plots of Volatile Composition in LFG of Different Origins

**Figure 1:**
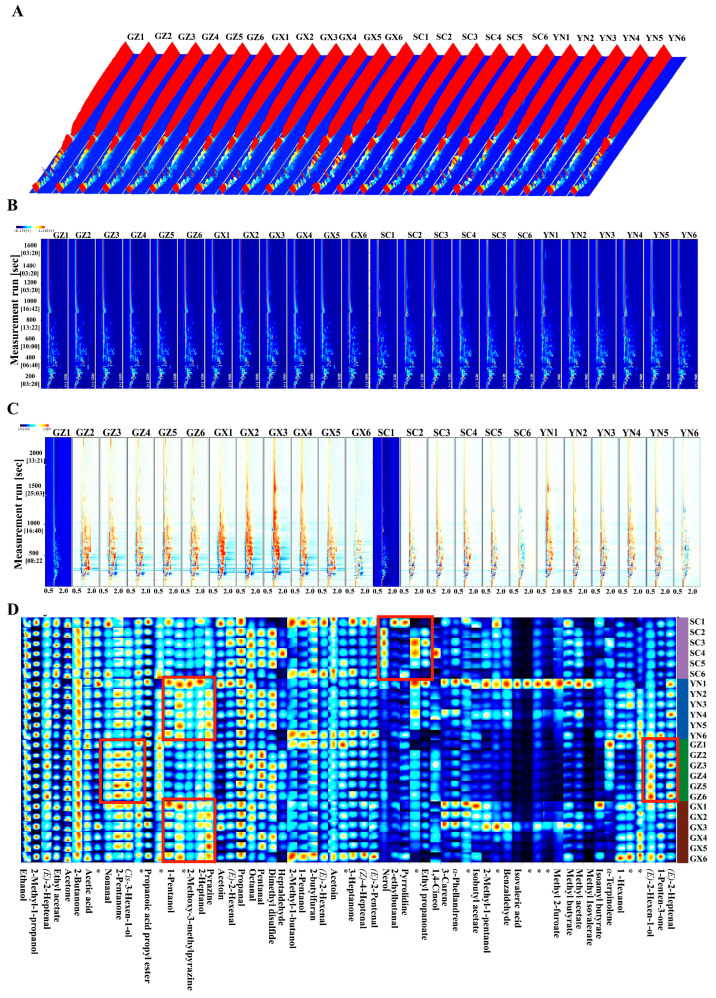
Volatile organic compounds (VOCs) in *Lysimachia foenum-graecum* Hance (LFG) samples from four geographical origins (Guangxi, GX; Guizhou, GZ; Sichuan, SC; Yunnan, YN) as determined by gas chromatography-ion mobility spectrometry (GC-IMS). (**A**) Three-dimensional topographic map of VOCs in LFG samples from the four regions. (**B**) Two-dimensional topographic chromatograms of VOCs in LFG samples from the four regions. (**C**) Differential comparison chromatogram showing VOC differences among the four regions. (**D**) Fingerprint profiles of VOCs in LFG samples from the four regions. Data were obtained from six independent replicates. * indicates the identified compounds. Compounds enclosed by the red frame exhibited higher signal intensities relative to the other production regions.

In this study, the experimental results of LFG samples from four geographical origins (Figure 1A) are displayed in the form of a three-dimensional map. The X-axis represents the ion mobility time of the compounds, the Y-axis denotes their gas chromatographic retention time, and the Z-axis reflects the peak intensity. The red vertical line at 1.0 on the X-axis corresponds to the normalized reaction ion peak (RIP) [19]. Compounds with a strong proton affinity may lead to a reduction in the RIP signal intensity [20]. The topographic map can vividly display the quantity and corresponding intensity of compounds in the LFG. In the map, the blue area serves as the background, the red peaks represent compounds with relatively high responses, and the white areas indicate low responses [21].

To better visualize the response intensity of compounds on a two-dimensional plane, the three-dimensional map was converted into a planar view (Figure 1B). In this view, blue represents the background, and each colored dot represents a compound. The color gradient from red to white reflects a decrease in response intensity from high to low. Upon examining the top-down view, it was observed that LFG samples from the four origins share similar compound profiles, with minor variations in their relative abundances. To further investigate these differences, reference maps from GZ1 and SC1 were used to generate a differential map (Figure 1C). The results indicate that the total compound content in samples from GX is slightly higher than that in GZ, and similarly, the total content in YN samples is slightly higher than that in SC.

### 3.2. Characteristic Fingerprint Analysis of Volatile Compounds in LFG from Different Origins Based on GC-IMS

To further investigate the potential differences among the LFG from the four production areas, we conducted a qualitative analysis of the results and drew a fingerprint map (Figure 1D). A total of 97 signal values were detected, and 51 compounds were identified (Table S1). Among them, there were 12 aldehydes, 10 esters, 10 alcohols, 7 ketones, and 12 others. There were also 46 unknown peaks. Notably, when the concentration of some compounds was too high, two peaks of monomer and dimer would appear [22]. The differences in the content of LFG compounds from different sources can be intuitively seen in the figure. Each column represents a compound, and each row represents a sample. The cells with red color indicate higher content, while those with blue indicate lower content [23]. We can see that in SC, the relative contents of Nerol and Ethyl propanoate were significantly higher than those in the other three regions. The relative contents of Cis-3-Hexen-1-ol, 2-Propanone, Nonanal, (E)-2-Hexen-1-ol, and (E)-2-Heptenal were relatively high in GZ. At the same time, it can be seen that the compounds Pyrazine, 2-Heptanol, 2-Methoxy-3-methylpyrazine, and 1-Pentanol were significantly higher in GX and YN compared to those in SC and GZ. These compounds contribute to the different flavor characteristics of LFG.

**Figure 2:**
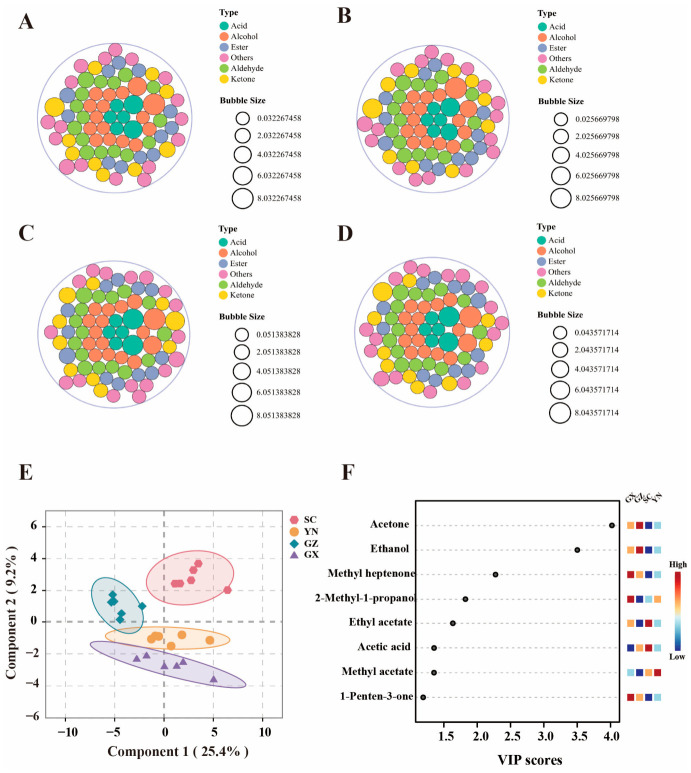
Statistical analysis of volatile organic compounds (VOCs) in *Lysimachia foenum-graecum* Hance (LFG) samples from four geographical origins (Guangxi, GX; Guizhou, GZ; Sichuan, SC; Yunnan, YN) as determined by gas chromatography-ion mobility spectrometry (GC-IMS). (**A**–**D**) Circle packing plots of VOCs profiles for (**A**) GX, (**B**) GZ, (**C**) SC, and (**D**) YN. Each circle represents a compound; circles of the same color denote the same chemical entity; bubble size indicates the relative peak volume magnitude. (**E**) Partial least squares discriminant analysis (PLS-DA) score plots of VOCs in LFG samples from the four regions. (**F**) Variable importance in projection (VIP) values derived from the GC-IMS-based PLS-DA. Data were obtained from six independent replicates.

To facilitate the identification of differences in LFG compounds across the production areas, the results were visualized using bubble stacked plots. Within these plots, compounds are grouped indicated by color, the number of circles of the same color represents the number of compounds in that class, each individual circle represents a single compound, and the size of the circle corresponds to its relative peak volume [24]. Analysis of the plots revealed distinct patterns: acids exhibited considerably larger relative peak volumes in the SC and YN samples (Figure 2A–D); similarly, esters showed significantly larger relative peak volumes in SC compared to the other three production areas.

### 3.3. Discrimination of LFG from Different Regions by PLS-DA Based on GC-IMS

To elucidate differences among the LFG samples from the four production areas, supervised orthogonal partial least squares-discriminant analysis PLS-DA models, combined with variable importance in projection VIP values, were applied to the compounds identified by GC-IMS. The PLS-DA model effectively differentiated the sample groups based on their chemical profiles and thereby pinpointed specific compounds responsible for the inter-group variations. (Figure 2E) [25]. Therefore, this model is adopted to distinguish the LFG from four different origins and to identify the significant variables that cause the differences [22]. A discernible separation trend among the LFG samples from the four origins was observed, suggesting subtle compositional differences. The VIP values derived from the PLS-DA model were used to analyze the LFG samples across origins and identify significant volatile flavor compounds. Compounds with VIP values > 1 are generally considered potential markers for discriminating between sample groups [26]. Consequently, 8 VOCs were screened (Figure 2F). These markers were acetone, ethanol, methyl heptenone, 2-methyl-1-propanol, ethyl acetate, acetic acid, methyl acetate, and 1-penten-3-one.

### 3.4. Quantitative Analysis of Volatile Compounds in LFG from Different Regions by GC-MS

**Figure 3:**
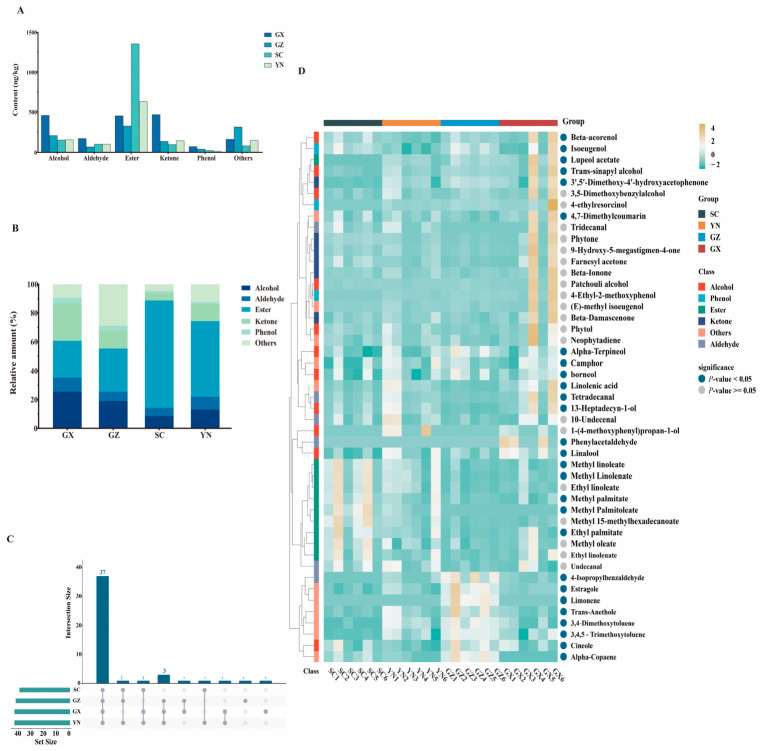
Volatile organic compounds (VOCs) in *Lysimachia foenum-graecum* Hance (LFG) samples from four geographical origins (Guangxi, GX; Guizhou, GZ; Sichuan, SC; Yunnan, YN) as determined by gas chromatography-mass spectrometry (GC-MS). (**A**) Content of different types of VOCs in LFG samples from the four regions. (**B**) Percentage composition of different types of VOCs. (**C**) UpSet plot showing the overlap and unique VOCs among the four regions. (**D**) Heatmap showing the relative abundance of VOCs in LFG samples from the four regions. Data were obtained from six independent replicates.

GC-MS identified a total of 47 volatile compounds (Table 1), including 11 alcohols, 6 aldehydes, 10 esters, 6 ketones, 3 phenols and 11 others. Significant regional differences were observed in 28 volatile compounds, particularly among esters and terpenoids. Methyl palmitoleate was detected exclusively in SC and YN, while limonene and α-copaene were unique to GZ, suggesting these compounds could serve as regional markers. Alpha -Terpineol (*p* = 0.000002) and methyl palmitoleate (*p* = 0.000004) showed the most pronounced variations. Notably, several differentially accumulated compounds, including linalool and cineole, are known aroma contributors, which aligns with the sensory evaluation results showing distinct aroma profiles among regions. These differences likely reflect environmental or genetic variation across the four production areas. Among the four production areas, GX and YN yielded the highest number of identified compounds, both at 44, while SC had the fewest at 40, and GZ had 43. Subsequent semi-quantitative analysis using an internal standard (Figure 3A,B) clearly showed that the ester content in SC was significantly higher than in the other three areas, reaching 1353 ng/kg. It was also evident that esters accounted for a relatively high proportion of the total volatile content in SC, representing 74%. In contrast, the proportions in GX, GZ, and YN were 25%, 29%, and 52%, respectively. Among these esters, methyl palmitate was present at a relatively high level. This compound possesses fruity and creamy aromas. Esters can impart plant-like fragrances [27]. In Baijiu (Chinese liquor), they work synergistically with other esters (such as ethyl hexanoate) to contribute fruity notes to the liquor body [28].

The percentage contents of ketone and alcohol compounds in SC were also significantly higher than those in the three production areas (Figure 3A). Phytone is a compound with a relatively high content among ketone compounds and can make the tea flavor more rich [29]. It is also an active component of the anti-cancer plant Vicia ochroleuca Ten [30]. It has anti-inflammatory, antioxidant and anti-tumor cell proliferation pharmacological effects [31]. Linalool, the compound with the highest proportion in the alcohol components, as a monoterpenol, has floral and fruity scents [32]. By observing the bar chart and combining it with the semi-quantitative results (Table 1), it is easy to see that trans-anethole has a higher content in GZ, which is a key aroma compound in the cooking process [33]. Its aroma characteristics are anise and sweet [34], and it has antioxidant and antibacterial capabilities [35].

There is a total of 37 compounds common to the four production areas (Figure 3C). In GZ, the contents of estragole, cineole, α-copaene, limonene, trans-anethol, and borneol are generally high. In GX, the contents of linolenic acid and tetradecanal are higher. The content of methyl palmitate is the lowest in both GZ and GX (Figure 3D). During the observation process, we found that the contents of different types of VOCs in GX are relatively good, and LFG is also widely used in GX. SC and YN have relatively high contents of ester components, and LFG itself is also one of the raw materials of Zhuyeqing Liquor. Ester compounds account for approximately 60% of the flavor substances in the liquor [36]. Therefore, it is speculated that the LFG from SC and YN would have better wine-making flavor. The contents of various compounds in GX are relatively high, so we speculate that these compounds would work best when used as raw materials for perfumes and cosmetics, while GZ might be more suitable for use as a cooking brine ingredient. In summary, the number and composition of VOCs in LFG from different regions are slightly different. Qualitative and quantitative analysis of these compounds is helpful in clarifying and identifying the unique flavor characteristics of different origins.

### 3.5. Discrimination of LFG from Different Origins by PLS-DA Based on GC-MS

**Figure 4:**
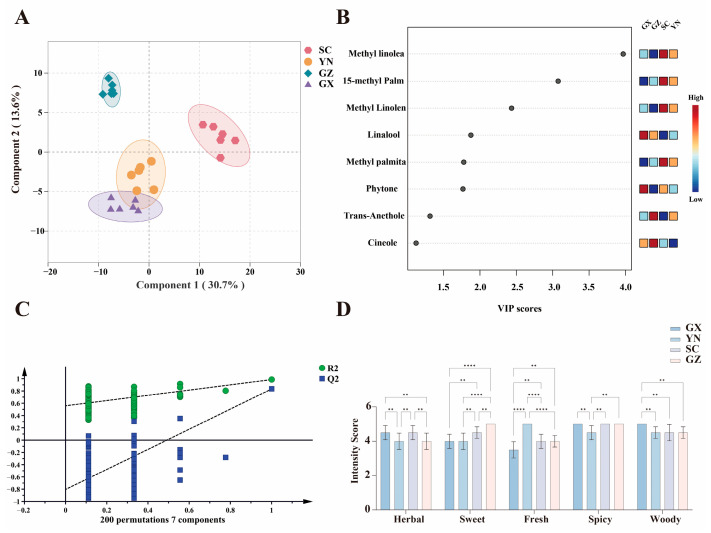
Statistical analysis of volatile organic compounds (VOCs) in *Lysimachia foenum-graecum* Hance (LFG) samples from four regions (Guangxi, GX; Guizhou, GZ; Sichuan, SC; Yunnan, YN) by gas chromatography-mass spectrometry (GC-MS). (**A**) PLS-DA score plots. (**B**) VIP values. (**C**) Permutation test plot. (**D**) Sensory evaluation scores for five aroma attributes (herbal, sweet, fresh, spicy, woody). Sensory data are mean ± SD (*n* = 10 evaluators). One-way ANOVA with Tukey’s post hoc test: herbal, GX and SC > YN and GZ (*p* < 0.01); sweet, GZ > all others (*p* < 0.0001), SC > GX and YN (*p* < 0.01); fresh, YN > all others (*p* < 0.0001), GX < SC and GZ (*p* < 0.01); spicy, YN < GX, SC, GZ (*p* < 0.01); woody, GX > YN, SC, GZ (*p* < 0.01).Asterisks in the figure denote significance levels: **, *p* < 0.01, ****, *p* < 0.0001.

To further clarify the differences among different production areas, PLS-DA was used to analyze the VOCs. As a supervised multivariate method, PLS-DA is characterized by its ability to perform regression analysis on systems with multiple independent and dependent variables. Compared with PCA, it provides better analytical performance and validity [37]. It was observed that there was a significant separation between groups (Figure 4A). Additionally, after 200 permutation tests, the regression line of Q2 intersected with the negative half-axis of the vertical axis, proving that the model did not overfit. The VIP evaluation can analyze the LFG of different production areas and identify important volatile flavor compounds [38]. Compounds with VIP > 1 were considered potential markers for distinguishing differences between samples (Figure 4B). A total of eight different components were screened out, namely methyl linoleate, methyl 15-methylhexadecanoate, methyl linolenate, linalool, methyl palmitate, phytone, trans-anethole, cineole. Based on the screening results, it is not difficult to find that there are many ester components, and methyl linoleate (VIP = 3.969), methyl 15-methylhexadecanoate (VIP = 3.074) and methyl linolenate (VIP = 2.434) are particularly important volatile compounds.

By screening the compounds with *p* < 0.05 and VIP > 1 (Table S2), 7 compounds were identified as the key differentiating compounds: methyl linoleate, methyl 15-methylhexadecanoate, methyl linolenate, linalool, methyl palmitate, trans-anethole, and cineole.

### 3.6. OAV Calculation Based on GC-MS and Sensory Evaluation

LFG contains a variety of aroma compounds. The aroma activity value can be calculated by the ratio of the concentration of the compounds to the odor threshold of the volatile flavor substances in water. It is an important indicator for evaluating the contribution of volatile compounds to the overall aroma of the substance. Generally, compounds with an OAV > 1 affect the overall aroma characteristics of the sample [39]. In this study, a total of 5 compounds with OAV > 1 were screened out (Table S3): linalool, borneol, estragole, undecanal, and β-Ionone.

Linalool is a common VOC in plants, and its aroma characteristics are a blend of fresh floral and citrus fruit scents [40]. It is also possible that through photosynthesis, linalool may impart a more intense tea aroma to tea leaves [41]. Its characteristics are a low odor threshold and sweet flavor [42]. Linalool has the highest content among alcohols, so it is hypothesized that linalool may give LFG a richer flavor profile, highlighting its citrus fruit aroma; moreover, the presence and content of linalool may also affect the release and retention time of the aroma, making it linger for a long time [43].

Borneol has a strong pine aroma, camphor scent and mint flavor [44]. Its presence can provide unique aroma characteristics for plants and emit a refreshing, cool and slightly woody fragrance [45]. The estragole compound has an aniseed and fennel-like aroma and has a significant impact on the aroma characteristics of plants [46]. It can interact with β-ionone to help form “woodsy”, “herbaceous”, “leafy” and “floral” flavors [47]. Estragole has a low content ratio in LFG, but its odor threshold is low, and it can be perceived even at lower concentrations [48], which is one of the reasons why it becomes a key aroma compound.

β-ionone and undecanal respectively possess floral and fruity aroma characteristics [49], which enhance the sweetness of tea and increase with the increase in concentration [50]. Therefore, it is hypothesized that these two compounds contribute fresh fruity and floral aromas to the aroma of LFG. It is notable that the ester chemical components account for a high proportion in LFG, but their OAV < 1. Thus, we speculate that the ester in LFG mainly interacts with other compounds to contribute to the aroma.

Sensory evaluation (Figure 4D) showed significant regional differences in certain attributes. GX had higher woody scores than YN, SC, and GZ (*p* < 0.05). YN had higher fresh scores (*p* < 0.0001). GZ had the highest sweet scores, followed by SC (*p* < 0.05). Herbal scores were higher in GX and SC (*p* < 0.05). Spicy scores were lower in YN (*p* < 0.05). Despite these differences, the overall aroma profiles across the four origins were similar, dominated by spicy and herbal notes. Consistent with OAV analysis, the aroma characteristics were not determined by a single compound but by the balance of multiple key aroma compounds.

### 3.7. Putative Metabolic Pathways of Key Volatile Compounds

To explore the possible formation routes of major volatile compounds in LFG, we referenced the KEGG Pathway and MetaCyc databases. Two compounds were found to have predicted pathways consistent with known plant and microbial metabolism (Figure S3).

In the terpenoid metabolism pathway, linalool is predicted to be synthesized from geranyl pyrophosphate (GPP) via linalool synthase, a typical reaction in terpenoid metabolism. In the amino acid metabolism pathway, 2-methyl-1-propanol (isobutanol) is predicted to derive from L-valine through sequential transamination, decarboxylation, and reduction via alcohol dehydrogenase [51].

### 3.8. Correlation Analysis of VOCs Determined by GC-IMS and GC-MS

**Figure 5:**
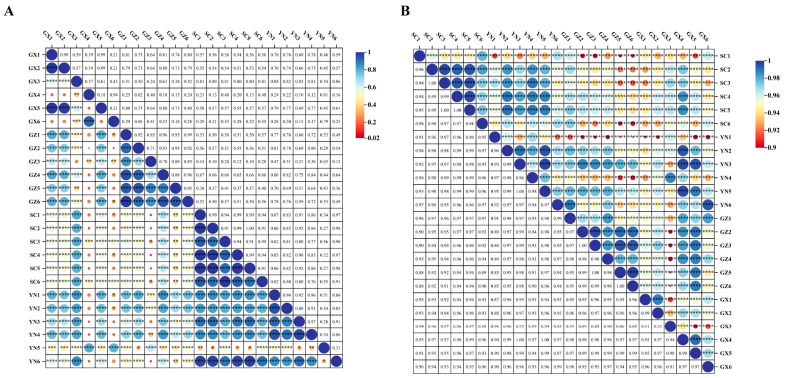
Cluster analysis of volatile organic compounds (VOCs) in *Lysimachia foenum-graecum* Hance (LFG) samples from four geographical origins (Guangxi, GX; Guizhou, GZ; Sichuan, SC; Yunnan, YN) as determined by gas chromatography-mass spectrometry (GC-MS) and gas chromatography-ion mobility spectrometry (GC-IMS). (**A**) Correlation plot based on GC-MS data. (**B**) Correlation plot based on GC-IMS data. Asterisks below the panel labels indicate the similarity level between samples, corresponding to the following statistical significance levels: ****, *p* ≤ 0.0001; ***, *p* ≤ 0.001; **, *p* ≤ 0.01; *, *p* ≤ 0.05. Data were obtained from six independent replicates.

Correlation analysis can quickly reveal the potential relationship between two variables [52]. The VOCs results of 24 batches of LFG from four production areas measured by GC-MS and GC-IMS were subjected to correlation analysis, and the data were visualized using the correlation heat map (Figure 5). The intensity of the circle color reflects the strength of the correlation; darker colors indicate a stronger correlation. Similarly, the number of asterisks on the map is proportional to the magnitude of the correlation [53]. There is a significant correlation between the products produced by YN and those produced by SC (Figure 5A); there is also a significant correlation between the samples SC and YN (Figure 5B), while the correlation between SC and GZ is relatively low. By integrating semi-quantitative and statistical analysis, it is evident that the VOCs in LFG vary with geographical origin. Moreover, the closer the geographical locations of the origins, the higher the correlation among their VOC profiles.

### 3.9. Effects of CORT and LFG on PC12 Cell Viability

**Figure 6:**
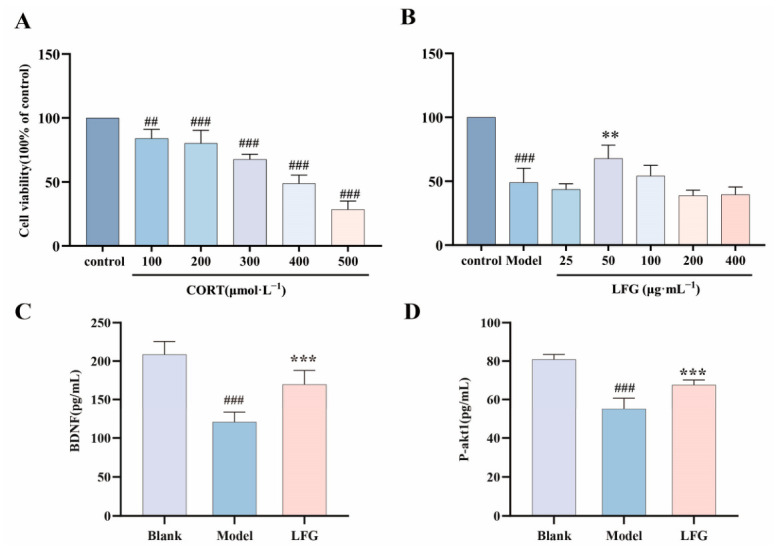
Protective effects of *Lysimachia foenum-graecum* Hance (LFG) on corticosterone (CORT)-induced injury in PC12 cells. (**A**) Effect of different concentrations of CORT on PC12 cell viability, measured by the Cell Counting Kit-8 (CCK-8) assay. (**B**) Effect of different concentrations of LFG on PC12 cell viability, measured by the CCK-8 assay. (**C**) Brain-derived neurotrophic factor (BDNF) content in PC12 cells. (**D**) Phosphorylated protein kinase B (p-Akt1) content in PC12 cells. Statistical significance: ** *p* < 0.01 and *** *p* < 0.001 indicate significant differences compared to the Model group; ^##^ *p* < 0.01 and ^###^ *p* < 0.001 indicate significant differences compared to the Control group.

Based on the CCK-8 results of CORT-induced PC12 cell injury (Figure 6A), we found that when the corticosterone concentration reached 400 μmol/L, the survival rate of PC12 cells significantly decreased to approximately 55% [54]. This indicates that this concentration can effectively induce the cell injury model, and was therefore selected as the optimal concentration for establishing the in vitro cell injury model.

Building upon this foundation, we further evaluated the impact of different dosages on cell survival within the CORT-induced injury model. Among multiple candidate doses, the 50 µg/mL treatment group exhibited the optimal cell survival rate (Figure 6B), significantly mitigating corticosterone-induced cellular damage. Therefore, we ultimately selected 50 µg/mL as the dosage for subsequent experiments to further investigate the protective mechanism of LFG against PC12 cell injury.

### 3.10. In the PC12 Cell Injury Model, LFG Reverses the Levels of BNDF and p-Akt1

We found that BDNF levels in the LFG-treated group showed a significant rebound compared to the model group (Figure 6C). This result suggests that the neuroprotective effects of LFG may be closely related to its upregulation of endogenous BDNF expression, thereby enhancing neurotrophic effects. As a major expressed neurotrophic factor, BDNF plays a crucial role in the development and maintenance of neural tissue [55]. It is commonly used in studies of traumatic nerve injury [56] and in regulating neurodegenerative diseases (including Alzheimer’s disease (AD), Parkinson’s disease (PD), and Huntington’s disease (HD) [57], affecting neuronal survival, synaptic plasticity, and cognitive function [58].

Upon binding of BDNF to its specific receptor TrkB, key signaling pathways such as PI3K/Akt and Ras/MAPK are activated, which predominantly promote cell survival and suppress apoptosis [59]. Activation of Akt1 subsequently leads to phosphorylation of CREB, thereby inducing the transcription of downstream target genes—including BDNF itself—forming a positive feedback loop that contributes to neuroprotection [60].

Compared with the model group (Figure 6D), LFG administration can significantly reverse the downregulation of p-Akt1 level induced by the corticosterone model. This finding is highly consistent with the previous observation of BDNF upregulation. Therefore, we speculate that it may be the restoration of BDNF levels that directly leads to the enhancement of AKT phosphorylation as the key downstream effector molecule. Additionally, some studies have shown that the activation of Akt1 may upregulate BDNF expression through CREB signaling [61]. Research has also found that curcumin can regulate the GSK3β/Wnt/β-catenin and CREB/BDNF pathways by targeting PI3K/Akt, thereby improving adult neurogenesis in AD mice [62]. This study found that LFG functions through the BDNF-TrkB-PI3K/Akt1 pathway, and the positive feedback regulatory mechanism between BDNF and AKT provides theoretical support for the neuroprotective effect of LFG.

## 4. Conclusions

In conclusion, a combined analytical approach employing GC-IMS and GC-MS was utilized to investigate VOCs in LFG samples sourced from four different geographical origins, enabling the successful characterization of inter-source compositional differences. The results demonstrated that esters, alcohols, and ketones were predominant compound classes, contributing significantly to the herbal, sweet, fresh, spicy, and woody aromatic profiles of LFG. Key aroma-active compounds identified included linalool, borneol, estragole, undecanal, and β-ionone. Multivariate statistical analysis using the PLS-DA model, along with VIP values, enabled the identification of 15 VOCs exhibiting significant inter-regional variation. These included acetone, ethanol, methylheptenone, 2-methyl-1-propanol, ethyl acetate, acetic acid, methyl acetate, 1-penten-3-one, methyl linoleate, methyl 15-methylhexadecanoate, methyl linolenate, linalool, methyl palmitate, trans-anethole, cineole. The number and composition of VOCs in LFG varied slightly across regions. In SC, ester content accounted for up to 74% of the total, suggesting its potential better suitability for producing liquor. As a designated region of origin of LFG, GX demonstrated higher overall compound contents compared to the other three regions. In vitro experiments demonstrate that LFG attenuates CORT-induced neurotoxicity in PC12 cells by restoring BDNF and p-Akt1 levels, providing a mechanistic basis for its therapeutic potential. This study provides a robust scientific foundation and empirical data to support LFG origin traceability and its application in the food industry, while also establishing a theoretical basis for the development of high-value, regionally distinctive products.

## Figures and Tables

**Table 1:** Volatile organic compounds (VOCs) detected in *Lysimachia foenum-graecum* Hance (LFG) samples from four geographical origins (Guangxi, GX; Guizhou, GZ; Sichuan, SC; Yunnan, YN) as analyzed by gas chromatography-mass spectrometry (GC-MS). Data are presented as mean ± SD (*n* = six independent replicates).

No.	Name	CAS	Formula	RI	RI *	*p*-Value	Count ng/kg			
							GX	GZ	SC	YN
	**Alcohol**									
1	Cineole *	470-82-6	C_10_H_18_O	1190	1193	0.002099	257.69 ± 9.64	473.88 ± 22.95	281.17 ± 22.64	89.72 ± 27.85
2	Linalool *	78-70-6	C_10_H_18_O	1110	1095	0.021590	887.43 ± 4.65	601.86 ± 18.55	414.99 ± 26.28	569.59 ± 164.48
3	Borneol	507-70-0	C_10_H_18_O	1158	1161	0.001066	153.74 ± 4.03	180.75 ± 74.35	116.77 ± 16.94	104.08 ± 35.32
4	Alpha-Terpineol	10482-56-1	C_10_H_18_O	1695	1697	0.000002	116.76 ± 1.42	180.56 ± 51.75	69.08 ± 4.7	88.55 ± 14.97
5	1-(4-methoxyphenyl)propan-1-ol	5349-60-0	C_10_H_14_O_2_	1317	1315	NA	10.19 ± 1.33	ND	ND	20.65 ± 4.52
6	β-acorenol	28400-11-5	C_15_H_26_O	1653	1649	0.000570	138.54 ± 16.73	104.43 ± 30.72	107.13 ± 16.22	32.56 ± 2.62
7	3,5-Dimethoxybenzylalcohol	705-76-0	C_9_H_12_O_3_	1620	1623	NA	253.84 ± 21.07	137.1 ± 48.56	82.09 ± 21.12	113.04 ± 8.91
8	Trans-sinapyl alcohol	20675-96-1	C_11_H_14_O_4_	1839	1842	0.003386	345.72 ± 41.78	184.1 ± 24.04	39.18 ± 4.24	111.25 ± 18.34
9	Phytol	150-86-7	C_20_H_40_O	2140	2145	NA	293.32 ± 30.2	109.55 ± 25.05	155.55 ± 44.66	143.64 ± 8.68
10	Patchouli alcohol	5986-55-0	C_15_H_26_O	1670	1666	NA	851.17 ± 68.7	116.5 ± 76.58	89.74 ± 25.96	52.68 ± 7.92
11	13-Heptadecyn-1-ol	56554-77-9	C_17_H_32_O	1962	1971	0.000823	270.34 ± 20.29	33.56 ± 24.49	134.27 ± 53.79	180.87 ± 19.06
	**Aldehyde**									
12	Phenylacetaldehyde	122-78-1	C_8_H_8_O	1192	1081	0.009550	21.7 ± 2.92	ND	ND	ND
13	Undecanal	112-44-7	C_11_H_22_O	1314	1309	NA	334.97 ± 36.12	224.87 ± 84.3	452.92 ± 69.41	371.45 ± 80.74
14	10-Undecenal	112-45-8	C_11_H_20_O	1294	1288	0.654820	298.42 ± 2.67	172.92 ± 65.42	243.95 ± 67.03	292.63 ± 39.59
15	4-Isopropylbenzaldehyde	122-03-2	C_10_H_12_O	1243	1246	0.000048	13.66 ± 0.41	147.15 ± 76.91	ND	20.21 ± 2.21
16	Tridecanal	10486-19-8	C_13_H_26_O	1527	1512	NA	148.36 ± 9.89	ND	80.09 ± 25.68	73.63 ± 18.23
17	Tetradecanal	124-25-4	C_14_H_28_O	1932	1933	0.000607	512.08 ± 42.65	164.03 ± 24.31	227.32 ± 51.86	274.33 ± 51.71
	**Ester**									
18	Methyl palmitate	112-39-0	C_17_H_34_O_2_	1930	1926	0.000685	615.62 ± 53.01	590.89 ± 83.62	2131.62 ± 398.28	879.31 ± 66.51
19	Methyl Palmitoleate	1120-25-8	C_17_H_32_O_2_	2240	2240	0.000004	ND	ND	267.51 ± 77.25	82.51 ± 7.27
20	Methyl 15-methylhexadecanoate	6929-04-0	C_18_H_36_O_2_	1793	1789	NA	420.24 ± 82.9	391.95 ± 60.29	2847.41 ± 595.19	908.54 ± 88.44
21	Ethyl palmitate	628-97-7	C_18_H_36_O_2_	2240	2243	0.009724	208.82 ± 28.64	81.78 ± 76.28	458.13 ± 39.09	192.29 ± 17.91
22	Methyl oleate	112-62-9	C_19_H_36_O_2_	2100	2102	NA	611.56 ± 48.9	428.82 ± 109.89	1063.7 ± 149.75	464.11 ± 69.58
23	Methyl linoleate *	112-63-0	C_19_H_34_O_2_	2100	2098	0.004180	1113.46 ± 98.64	1008.58 ± 152.28	3602.6 ± 429.05	1896.04 ± 205.92
24	Methyl Linolenate	301-00-8	C_19_H_32_O_2_	2115	2108	0.001699	659.97 ± 50.36	385.92 ± 319.48	1921.8 ± 214.86	1069.68 ± 75.4
25	Ethyl linoleate	544-35-4	C_20_H_36_O_2_	2167	2162	0.007764	293.22 ± 17.98	205.81 ± 66.28	848.31 ± 248.25	478.06 ± 85.95
26	Ethyl linolenate	1191-41-9	C_20_H_34_O_2_	2198	2201	NA	314.29 ± 25.9	132.52 ± 43.98	397.79 ± 156.53	177.16 ± 31.94
27	Lupeol acetate	1617-68-1	C_32_H_52_O_2_	2980	2987	0.000466	105.92 ± 13.52	58.34 ± 16.69	ND	34.5 ± 9.66
	**Ketone**									
28	Beta-Damascenone	6901-97-9	C_13_H_20_O	1426	1421	NA	146.64 ± 15.75	32.94 ± 12.28	40.67 ± 6.52	50.56 ± 2.53
29	β-Ionone	14901-07-6	C_13_H_20_O	1490	1488	NA	190.2 ± 21.51	55.25 ± 17.93	61.28 ± 17.39	62.69 ± 5.7
30	Acetosyringone	2478-38-8	C_10_H_12_O_4_	1740	1742	NA	458.29 ± 78.72	437.11 ± 54.5	134.66 ± 15.01	237.27 ± 35.39
31	9-Hydroxy-5-megastigmen-4-one	27185-79-1	C_13_H_22_O_2_	1960	1966	NA	290.87 ± 19.51	75.77 ± 19.61	109.5 ± 27.81	95.86 ± 3.88
32	Phytone	502-69-2	C_18_H_36_O_2_	2080	2081	NA	2973.51 ± 129.9	653.77 ± 145.24	1465.45 ± 227.8	776.55 ± 51.59
33	Farnesyl acetone	1117-52-8	C_18_H_30_O	1920	1916	NA	660.1 ± 73.9	132.04 ± 56.45	405.9 ± 21.97	178.79 ± 9.29
	**Phenol**									
34	4-ethylresorcinol	2896-60-8	C_8_H_10_O_2_	1340	1334	NA	151.1 ± 8.17	62.89 ± 8.63	ND	32.73 ± 16.94
35	4-Ethyl-2-methoxyphenol	2785-89-9	C_9_H_12_O_2_	1300	1303	NA	398.71 ± 23.99	74.06 ± 26.77	ND	37.85 ± 5.19
36	Isoeugenol	579-60-2	C_10_H_12_O_2_	1500	1499	0.000001	190.76 ± 16.65	260.59 ± 53.21	234.06 ± 60.06	79.3 ± 8.15
	**Others**									
37	(*E*)-Methyl isoeugenol	93-16-3	C_11_H_14_O_2_	1773	1768	NA	259.76 ± 31.5	90.59 ± 25.69	69.68 ± 5.4	71.88 ± 9.9
38	Limonene	5989-27-5	C_10_H_16_	1020	1018	0.0000007	ND	99.52 ± 6.44	ND	ND
39	Camphor	464-48-2	C_10_H_16_O	1140	1145	0.028558	90.03 ± 6.11	90.75 ± 20.06	54.39 ± 12.43	80.8 ± 24.44
40	Estragole	140-67-0	C_10_H_12_O	1190	1196	0.000000	83.6 ± 0.73	308.11 ± 109.57	38.19 ± 3.04	71.62 ± 52.13
41	Alpha-Copaene	3856-25-5	C_15_H_24_	1360	1362	0.0000002	ND	152.14 ± 47.15	55.99 ± 15.85	13.76 ± 1.52
42	3,4-Dimethoxytoluene	494-99-5	C_9_H_12_O_2_	1240	1233	0.000098	27.72 ± 1.02	101.63 ± 26.23	3.74 ± 0.17	46.62 ± 7.22
43	Trans-Anethole	4180-23-8	C_10_H_12_O	1280	1284	0.000003	434.43 ± 27.34	1863.15 ± 107.56	295.35 ± 77.18	719.35 ± 160.5
44	3,4,5—Trimethoxytoluene	6443-69-2	C_10_H_14_O_3_	1420	1421	0.000074	185.71 ± 10.85	355.16 ± 36.31	94.13 ± 11.6	149.03 ± 25.82
45	Neophytadiene	504-96-1	C_20_H_38_	1915	1922	NA	109.53 ± 17.98	34.77 ± 31.9	43.25 ± 9.24	49.85 ± 9.03
46	6,7-Dimethoxycoumarin	120-08-1	C_11_H_10_O_4_	2020	2028	NA	94.66 ± 9.66	42.42 ± 11.59	57.65 ± 3.91	43.33 ± 10.74
47	Linolenic acid	463-40-1	C_18_H_30_O_2_	2110	2115	0.000003	290.62 ± 25.48	47.31 ± 15.69	83.45 ± 7.62	186.02 ± 24.23

RI, Retention Index on DB-WAX; RI *, Retention Index for the NIST 2017database; ND, indicates for the compound not been identified. Cineole *, Linalool * and Methyl linoleate * were identified by comparison with reference substances; NA, not significant among regions (*p* > 0.05).

## Data Availability

The data supporting the findings of this study are available in Mendeley Data at https://data.mendeley.com/datasets/txb7cnbvjr/1 (accessed on 18 March 2026).

## Supplementary Materials

The following supporting information can be downloaded at: https://www.mdpi.com/article/10.3390/foods15122245/s1, Table S1: The volatile organic compounds detected in different origin LFG analyzed by GC-IMS. Table S2: Odor description of the VOCs with a VIP > 1 and *p* < 0.05 was detected by GC-IMS and GC-MS. Table S3: The key aroma compounds with OAV ≥ 1 in GC-MS. Figure S1: Representative GC-IMS two-dimensional topographic map for VOC identification in *Lysimachia foenum-graecum* Hance (LFG) samples. Data were obtained from three independent replicates. Figure S2: Representative GC-MS total ion current (TIC) chromatogram of VOCs in a *Lysimachia foenum-graecum* Hance (LFG) sample. Data were obtained from three independent replicates. Figure S3: Putative biosynthetic pathways of linalool and 2-methyl-1-propanol. The upper panel shows the terpenoid metabolism pathway, with linalool putatively synthesized from geranyl pyrophosphate via linalool synthase. The lower panel shows the amino acid metabolism pathway, with 2-methyl-1-propanol putatively derived from L-valine via transamination, decarboxylation, and reduction. These speculative pathways are based on KEGG and MetaCyc databases and await experimental confirmation. Figure S4: Representative EI mass spectrum of linalool identified in *Lysimachia foenum-graecum* Hance (LFG) samples. The spectrum was matched against the NIST library with a match factor of 900.
