# Supplementary material for: GC-MS and GC-IMS Based Metabolomics Combined with Cellular Assays to Characterize Volatile Compounds and Pharmacological Activity of Lysimachia foenum-graecum Hance from Different Origins
Source: Foods. 2026 Jun 22;15(12):2245. doi: 10.3390/foods15122245 (PMC13298156; doi:10.3390/foods15122245)
Supplement: Supplementary file 1 [file foods-15-02245-s001.zip › Figure S2.pdf]

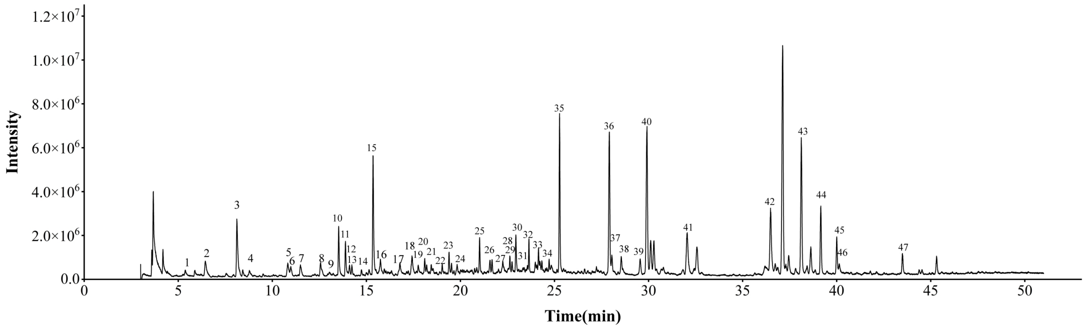

Figure S2. Representative GC-MS total ion current (TIC) chromatogram of VOCs in a *Lysimachia foenum-graecum* Hance (LFG) sample. Data were obtained from three independent replicates.
