# Supplementary material for: GC-MS and GC-IMS Based Metabolomics Combined with Cellular Assays to Characterize Volatile Compounds and Pharmacological Activity of Lysimachia foenum-graecum Hance from Different Origins
Source: Foods. 2026 Jun 22;15(12):2245. doi: 10.3390/foods15122245 (PMC13298156; doi:10.3390/foods15122245)
Supplement: Supplementary file 1 [file foods-15-02245-s001.zip › Figure S3.pdf]

.

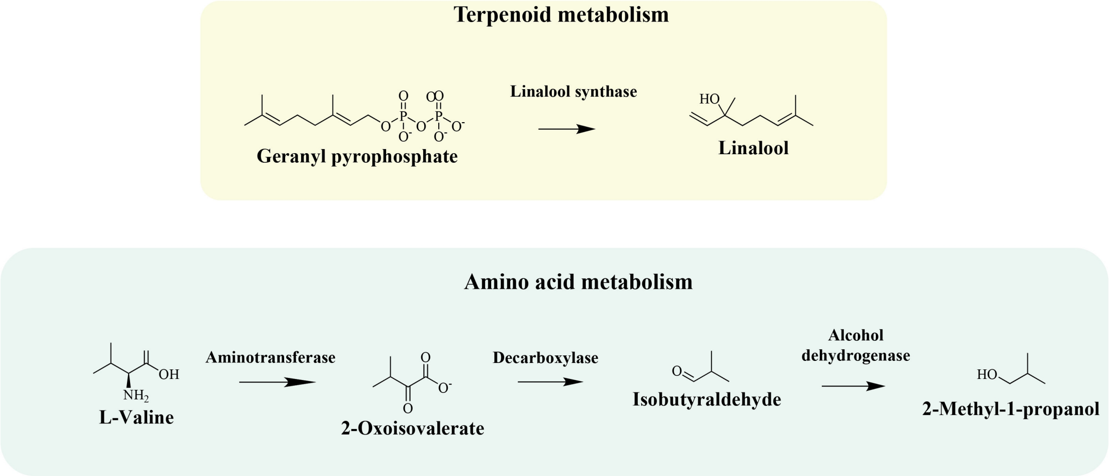

Figure S3. Putative biosynthetic pathways of linalool and 2-methyl-1-propanol.

The upper panel shows the terpenoid metabolism pathway, with linalool putatively synthesized from geranyl pyrophosphate via linalool synthase. The lower panel shows the amino acid metabolism pathway, with 2-methyl-1-propanol putatively derived from L-valine via transamination, decarboxylation, and reduction. These speculative pathways are based on KEGG and MetaCyc databases and await experimental confirmation.
