# Supplementary material for: GC-MS and GC-IMS Based Metabolomics Combined with Cellular Assays to Characterize Volatile Compounds and Pharmacological Activity of Lysimachia foenum-graecum Hance from Different Origins
Source: Foods. 2026 Jun 22;15(12):2245. doi: 10.3390/foods15122245 (PMC13298156; doi:10.3390/foods15122245)
Supplement: Supplementary file 1 [file foods-15-02245-s001.zip › Figure S4.pdf]

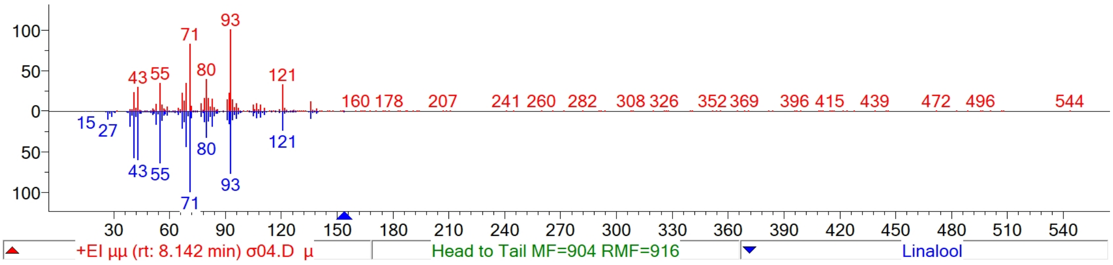

Figure S4. Representative EI mass spectrum of linalool identified in *Lysimachia foenum-graecum* Hance (LFG) samples. The spectrum was matched against the NIST library with a match factor of 900.
