# Supplementary material for: GC-MS and GC-IMS Based Metabolomics Combined with Cellular Assays to Characterize Volatile Compounds and Pharmacological Activity of Lysimachia foenum-graecum Hance from Different Origins
Source: Foods. 2026 Jun 22;15(12):2245. doi: 10.3390/foods15122245 (PMC13298156; doi:10.3390/foods15122245)
Supplement: Supplementary file 1 [file foods-15-02245-s001.zip › Table S1.pdf]

Table S1.

The volatile organic compounds detected in different origin LFG analyzed by GC-IMS.

No.	Name	CAS	Formula	MW	Retention Index (RI)	RI*	Rt [sec]	Dt [a.u.]
	**Alcohol**							
1	Nerol	106-25-2	$C_{10}H_{18}O$	154	1804	1797	2068.815	1.22862
2	*(E)*-2-Hexen-1-ol	928-95-0	$C_6H_{12}O$	100	1416	1416	816.616	1.33653
3	*Cis*-3-Hexen-1-ol	928-96-1	$C_6H_{12}O$	100	1390	1382	766.619	1.51511
4	1 -Hexanol	111-27-3	$C_6H_{14}O$	102	1364	1355	720.762	1.32658
5	2-Methyl-1-pentanol	105-30-6	$C_6H_{14}O$	102	1311	1293	635.34	1.28707
6	2-Heptanol	543-49-7	C7H16O	116	1333	1320	668.598	1.38095
7	*(E)*-2-Hexenal	6728-26-3	$C_6H_{10}O$	98	1218	1216	486.223	1.18569
8	2-Methyl-1-butanol	137-32-6	$C_5H_{12}O$	88	1202	1208	464.005	1.23214
9	2-Methyl-1-propanol	78-83-1	$C_4H_{10}O$	74	1091	1092	317.378	1.3615
10	Ethanol*	64-17-5	C_2H_6O	46.1	935	932	196.068	1.13306
	**Aldehyde**							
11	Benzaldehyde	100-52-7	C_7H_6O	106	1548	1553	1095.616	1.14656
12	Nonanal	124-19-6	$C_9H_{18}O$	142	1391	1391	769.188	1.47342
13	*(E)*-2-Heptenal	18829-55-5	C7H12O	112	1331	1334	665.826	1.2636

No.	Name	CAS	Formula	MW	Retention Index (RI)	RI*	Rt [sec]	Dt [a.u.]
14	Octanal	124-13-0	$C_8H_{16}O$	128	1293	1289	607.272	1.39702
15	*(Z)*-4-Heptenal	6728-31-0	$C_7H_{12}O$	112	1242	1240	522.335	1.1535
16	*(E)*-2-Hexenal	6728-26-3	$C_6H_{10}O$	98	1218	1216	486.223	1.18569
17	*(E)*-2-Pentenal	1576-87-0	C_5H_8O	84	1130	1127	363.263	1.36569
18	Heptaldehyde	111-71-7	$C_7H_{14}O$	114	1180	1184	482.679	1.68868
19	Pentanal	110-62-3	$C_5H_{10}O$	86	980	979	242.565	1.18869
20	Propanal	123-38-6	C_3H_6O	58	804	798	134.417	1.14994
21	2-Methyl-2-propenal	78-85-3	C_4H_6O	70	900	888	177.234	1.05847
22	2-Ethylbutanal	97-96-1	$C_6H_{12}O$	100	1023	1018	271.857	1.20869
	**Ester**							
23	Methyl 2-furoate	611-13-2	$C_6H_6O_3$	126	1566	1563	1171.605	1.14763
24	Isoamyl butyrate	106-27-4	$C_9H_{18}O_2$	158	1260	1259	456.344	1.36488
25	Methyl butyrate	623-42-7	$C_5H_{10}O_2$	102	985	982	226.674	1.43444
26	Ethyl acetate	141-78-6	C4H8O2	88	884	888	169.423	1.34356
27	Methyl acetate*	79-20-9	C3H6O2	74	854	845	155.35	1.19994
28	Isobutyl acetate	110-19-0	$C_6H_{12}O_2$	116	1042	1047	271.302	1.23471
29	Ethyl propanoate	105-37-3	$C_5H_{10}O_2$	102	959	953	210.72	1.45931

No.	Name	CAS	Formula	MW	Retention Index (RI)	RI*	Rt [sec]	Dt [a.u.]
30	Methyl isovalerate	556-24-1	$C_6H_{12}O_2$	116	1018	1018	251.819	1.53904
31	Methyl acrylate	96-33-3	$C_4H_6O_2$	86	948	938	210.029	1.27139
32	Propanoic acid propyl ester	106-36-5	$C_6H_{12}O_2$	116	1051	1044	306.338	1.57504
	**Ketone**							
33	Methyl heptenone	110-93-0	$C_8H_{14}O$	126	1290	1284	691.321	1.18404
34	Acetoin	513-86-0	$C_4H_8O_2$	88	1293	1284	608.735	1.05799
35	3-Heptanone	106-35-4	$C_7H_{14}O$	114	1170	1161	439.651	1.22669
36	1-Penten-3-one	1629-58-9	C_5H_8O	84	1024	1019	256.691	1.31915
37	Acetone	67-64-1	C_3H_6O	58	821	819	145.42	1.12045
38	2-Pentanone	107-87-9	$C_5H_{10}O$	86	984	981	225.975	1.37549
39	2-Butanone	78-93-3	C_4H_8O	72	923	914	195.375	1.24661
	**Others**							
40	Isovaleric acid	503-74-2	$C_5H_{10}O_2$	102	1688	1688	1568.229	1.21844
41	Acetic acid*	64-19-7	$C_2H_4O_2$	60	1454	1465	894.75	1.05172
42	Rose oxide	16409-43-1	$C_{10}H_{18}O$	154	1353	1350	718.688	1.35763
43	2-Methoxy-3-methylpyrazine	2847-30-5	$C_6H_8N_2O$	124	1321	1320	668.272	1.55472
44	α-terpinolene	586-62-9	$C_{10}H_{16}$	136	1281	1283	587.5	1.22584

No.	Name	CAS	Formula	MW	Retention Index (RI)	RI*	Rt [sec]	Dt [a.u.]
45	α-Phellandrene	99-83-2	$C_{10}H_{16}$	136	1170	1167	430.187	1.69105
46	3-Carene	13466-78-9	$C_{10}H_{16}$	136	1151	1152	392.279	1.21485
47	1-Penten-3-one	1629-58-9	C_5H_8O	84	1024	1019	256.691	1.31915
48	Dimethyl disulfide	624-92-0	$C_2H_6S_2$	94	1082	1077	280.149	1.14054
49	2-Butylfuran	4466-24-4	$C_8H_{12}O$	124	1130	1123	374.513	1.18276
50	Pyrrolidine	123-75-1	C_4H_9N	71	1025	1021	274.034	1.04596
51	Pyrazine	290-37-9	$C_4H_4N_2$	80	1215	1212	542.599	1.28503

Note:

MW, Molecular Weight

RI, Retention Index on DB-WAX.

RI*, Retention Index for the NIST17.0 database

Ethanol*, Acetic acid* and Methyl acetate* were identified by comparison with reference substances.

Rt [sec], Retention Time

Dt [a.u.], Drift Time
