# Supplementary material for: GC-MS and GC-IMS Based Metabolomics Combined with Cellular Assays to Characterize Volatile Compounds and Pharmacological Activity of Lysimachia foenum-graecum Hance from Different Origins
Source: Foods. 2026 Jun 22;15(12):2245. doi: 10.3390/foods15122245 (PMC13298156; doi:10.3390/foods15122245)
Supplement: Supplementary file 1 [file foods-15-02245-s001.zip › Table S2.pdf]

Table S2.

Odor description of the VOCs with a VIP＞1and P＜0.05 was detected by GC-IMS and GC-MS.

	VIP	*P*-value
**GC-IMS**		
Acetone	4.0214	-
Ethanol	3.5019	-
Methylheptenone	2.2749	-
2-Methyl-1-propanol	1.8234	-
Ethyl acetate	1.6347	-
Acetic acid	1.3529	-
Methyl acetate	1.3522	-
1-Penten-3-one	1.1895	-
**GC-MS**		
Methyl linoleate	3.9696	0.0041
15-methyl Palmitic Acid methyl ester	3.0748	0.0018
Methyl Linolenate	2.4348	0.0016
Linalool	1.8750	0.0215
Methyl palmitate	1.7771	0.0006
Trans-Anethole	1.3131	0.0003
Cineole	1.1211	0.0020
