# Supplementary material for: GC-MS and GC-IMS Based Metabolomics Combined with Cellular Assays to Characterize Volatile Compounds and Pharmacological Activity of Lysimachia foenum-graecum Hance from Different Origins
Source: Foods. 2026 Jun 22;15(12):2245. doi: 10.3390/foods15122245 (PMC13298156; doi:10.3390/foods15122245)
Supplement: Supplementary file 1 [file foods-15-02245-s001.zip › Table S3.pdf]

Table S3.

The key aroma compounds with OAV⩾1 in GC-MS

Numbers	Volatile compounds	Odor descriptions*	Odor threshold in water (μg/L)#	OAV			
				GX	GZ	SC	YN
1	Linalool	Floral, citrus	0.580	1.530	1.038	1.015	1.098
2	borneol	Pine fragrance, camphor, mint	0.080	1.922	2.259	1.460	1.301
3	Estragole	Fennel,Dill	0.020	4.180	15.405	2.291	4.297
4	Undecanal	Rose, woody, orange peel	0.300	1.340	1.050	1.510	1.238
5	β-Ionone	Violet,Earthy,Woody	0.021	9.057	2.631	2.918	2.985

*Odor description found in the literature with database (Flavornet; The LRI and Odour Database).
#All odor thresholds were obtained from: 'Odour & Flavour Detection Thresholds in Water (In Parts per Billion, lg/L) (http://www.leffingwell.com)(https://doi.org/10.1016/j.foodchem.2021.131933).
